# Brief Report: Diagnostic Accuracy of Oral Mucosal Transudate Tests Compared with Blood-Based Rapid Tests for HIV Among Children Aged 18 Months to 18 Years in Kenya and Zimbabwe

**DOI:** 10.1097/QAI.0000000000002146

**Published:** 2019-08-16

**Authors:** Chido Dziva Chikwari, Irene N. Njuguna, Jillian Neary, Crissi Rainer, Belinda Chihota, Jennifer A. Slyker, David A. Katz, Dalton C. Wamalwa, Laura Oyiengo, Tsitsi Bandason, Grace McHugh, Ethel Dauya, Hilda Mujuru, Kearsley A. Stewart, Grace C. John-Stewart, Rashida A. Ferrand, Anjuli D. Wagner

**Affiliations:** a Clinical Research Department, London School of Hygiene and Tropical Medicine, London, United Kingdom;; b Biomedical Research and Training Institute, Harare, Zimbabwe;; c Department of Epidemiology, University of Washington, Seattle, WA;; d Research and Programs, Kenyatta National Hospital, Nairobi, Kenya;; e Department of Global Health, University of Washington, Seattle, WA;; f Duke Global Health Institute, Duke University, Durham, NC;; g Centre for Infectious Disease Research in Zambia, Lusaka, Zambia;; h Department of Medicine, Division of Allergy and Infectious diseases, School of Medicine, University of Washington, Seattle, WA;; i Department of Paediatric and Child Health, University of Nairobi, Nairobi, Kenya;; j National AIDS & STI Control Programme, Ministry of Health, Nairobi, Kenya;; k University of Zimbabwe College of Health Sciences, Harare, Zimbabwe; and; l Departments of Medicine and Pediatrics, University of Washington, Nairobi, Kenya.

**Keywords:** HIV, children, pediatric, oral HIV testing, diagnostic, saliva HIV testing

## Abstract

Gaps persist in HIV testing for children who were not tested in prevention of mother-to-child HIV transmission programs. Oral mucosal transudate (OMT) rapid HIV tests have been shown to be highly sensitive in adults, but their performance has not been established in children.

**Methods:**

Antiretroviral therapy-naive children aged 18 months to 18 years in Kenya and Zimbabwe were tested for HIV using rapid OraQuick ADVANCE Rapid HIV-1/2 Antibody test on oral fluids (OMT) and blood-based rapid diagnostic testing (BBT). BBT followed Kenyan and Zimbabwean national algorithms. Sensitivity and specificity were calculated using the national algorithms as the reference standard.

**Results:**

A total of 1776 children were enrolled; median age was 7.3 years (interquartile range: 4.7–11.6). Among 71 children positive by BBT, all 71 were positive by OMT (sensitivity: 100% [97.5% confidence interval (CI): 94.9% to 100%]). Among the 1705 children negative by BBT, 1703 were negative by OMT (specificity: 99.9% [95% CI: 99.6% to 100.0%]). Due to discrepant BBT and OMT results, 2 children who initially tested BBT-negative and OMT-positive were subsequently confirmed positive within 1 week by further tests. Excluding these 2 children, the sensitivity and specificity of OMT compared with those of BBT were each 100% (97.5% CI: 94.9% to 100% and 99.8% to 100%, respectively).

**Conclusions:**

Compared to national algorithms, OMT did not miss any HIV-positive children. These data suggest that OMTs are valid in this age range. Future research should explore the acceptability and uptake of OMT by caregivers and health workers to increase pediatric HIV testing coverage.

## INTRODUCTION

The HIV pandemic has heavily affected children with over 1.8 million children (<15 years) living with HIV and 180,000 newly infected in 2017.^1^ Prompt diagnosis and initiation on antiretroviral therapy (ART) is associated with decreased morbidity and mortality^2,3^ and improved developmental outcomes^4,5^; however, gaps remain in diagnosis, particularly among older children and adolescents.^6^

World Health Organization (WHO) recommendations endorse rapid antibody-based HIV tests for diagnosis of individuals >18 months.^7^ Blood-based HIV tests (BBT) are used globally. In addition, oral mucosal transudate (OMT) rapid HIV tests allow for sample collection that is less invasive, are more acceptable to clients, poses fewer risks to health care workers (HCW), and may increase testing uptake.^8–10^

The Food and Drug Administration approved the OraQuick OMT in 2004 for testing by health providers for individuals >12 years.^11^ In 2016, the OraQuick HIV Self-Test received WHO prequalification and it is now recommended by WHO as a screening test for HIV.^12^ OMT has high sensitivity and specificity in detecting HIV antibodies in adults and older adolescents.^7,10^ A meta-analysis comparing OMT with BBT in adults reported a pooled sensitivity of 98.0% and specificity of 99.7% for OMT.^10^ OMT has not been validated in children.

We evaluated the diagnostic performance of OMT compared with routine BBT in children and adolescents aged 18 months to 18 years in Kenya and Zimbabwe.

## METHODS

### Setting and Participants

This analysis includes pooled data from 2 studies in Kenya and Zimbabwe that include parallel point-of-care diagnostic OMT and BBT to assess sensitivity and specificity of OMT among children and adolescents. Data were combined to increase precision of sensitivity and specificity estimates, as the number of newly diagnosed HIV-positive children in both settings has reduced with the scale-up of pediatric HIV prevention and treatment programs.

#### Zimbabwe

This analysis was nested within the “Bridging the Gap in HIV Testing and Care for Children in Zimbabwe” (B-GAP Project) whose aim is to evaluate index-linked testing for pediatric case detection. Study participants were children and adolescents of unknown HIV status, aged 2–18 years, attending any health services in the participating hospitals and primary health care clinics.

#### Kenya

The “Saliva Testing and Video Information to Expand Uptake of Pediatric HIV Testing” (STEP-UP) study enrolled children aged 18 months to 12 years. Two recruitment streams were used. First, children of HIV-positive adults attending HIV clinics who were tested for HIV within a randomized controlled trial of financial incentives for index case testing (FIT trial; NCT03049917^13^) were recruited after determining HIV status using BBT within the trial. Second, children from outpatient clinics were recruited after HIV testing using BBT within routine testing; here children who tested BBT positive were oversampled.

### Procedures

#### Zimbabwe

Testing followed the national algorithm^14^: first, BBT by Determine (Alere Determine HIV-1/2 Ag/Ab Combo; Abbott, Chicago, IL) (fourth generation), followed by First Response (First Response HIV-1-2; Premier Medical Corporation Ltd, Kachigam, India) (third generation), if Determine was reactive. In the case of 2 reactive BBTs, the same 2 BBTs were performed by a different provider to confirm a positive diagnosis. In the case of discordant BBTs, both tests were repeated. If discordance persisted, a third test, CHEMBIO was performed (CHEMBIO HIV 1/2 STAT-PAK Assay; CHEMBIO Diagnostic Systems, Inc., New York, NY). If this third test was positive, the result was reported as inconclusive and a retest conducted in 14 days. OMT was conducted by clinic staff blinded to BBT results.

#### Kenya

The national algorithm mirrored that in Zimbabwe with the following exceptions: the Determine HIV test was third instead of fourth generation and DNA PCR from dry blood spot specimens was the third test and was considered conclusive.^14–16^ In addition BBT was conducted by research staff for those enrolled in the FIT trial and non-research staff for those enrolled from routine testing points. Research staff performed OMT and were not blinded to BBT results.

The reference standard used for our study was the HIV status as per the national algorithim of each country.

#### OMT

In Zimbabwe and Kenya, OMT sample collection and processing was performed bedside by qualified HIV testing lay providers who are typically lower than nurse level providers and are responsible for HIV testing in both countries. The qualification for these providers is a standard national training for HIV services conducted over 2 weeks. Testing was conducted according to manufacturer details (OraQuick ADVANCE Rapid HIV-1/2 Antibody Test; OraSure Technologies, Inc., Bethlehem, PA), whereby the research staff collected an oral fluid sample from the participants by running the test device between the lips and outer gums of the client once on top and once at the bottom and then place the test device pad directly into the reaction fluid immediately after collection.^17^ OMT results were read once between 20 and 40 minutes in Zimbabwe, and twice in Kenya at both 20 and 40 minutes to assess test performance at the lower and upper recommended times. OMT results were not shared with caregivers, because the test was undergoing validation.

#### Statistical Analysis

Data were analyzed using STATA 14 (StataCorp, College Station, TX). Sensitivity was calculated by dividing the number of OMT and BBT-positive children by the number of BBT-positive children. Specificity was calculated by dividing the number of OMT and BBT-negative children by the number of BBT-negative children. Positive predictive value (PPV) and negative predictive value (NPV) were calculated in the Zimbabwean cohort by dividing the number with both positive OMT and BBT by all the positive OMT tests (PPV) and by dividing the number with both negative OMT and BBT results by the total negative by OMT (NPV). PPV and NPV were not calculated in the Kenyan cohort, because positive children were oversampled. Ninety-five percent (95%) or 97.5% (when the estimate was 100%) confidence intervals (CIs) were calculated using a binomial distribution. Stability of the test results using result interpretation pictures from the manufacturer was described in Kenya.

#### Ethics

Adolescents ≥16 gave independent written informed consent without parental/guardian consent. Parents/guardians of children aged 18 months–15 years provided written consent; adolescents 13–15 years signed a paragraph within the parental consent form to give their assent, whereas children 7–12 years signed a separate assent document, which was optional in Kenya. B-GAP received approval from the Biomedical Research and Training Institute, the Medical Research Council of Zimbabwe and institutional review boards at Duke University and the London School of Hygiene and Tropical Medicine. The Kenyan study received approval from the Kenyatta National Hospital Ethics and Research Committee and the University of Washington Institutional Review Board.

**TABLE 1.**
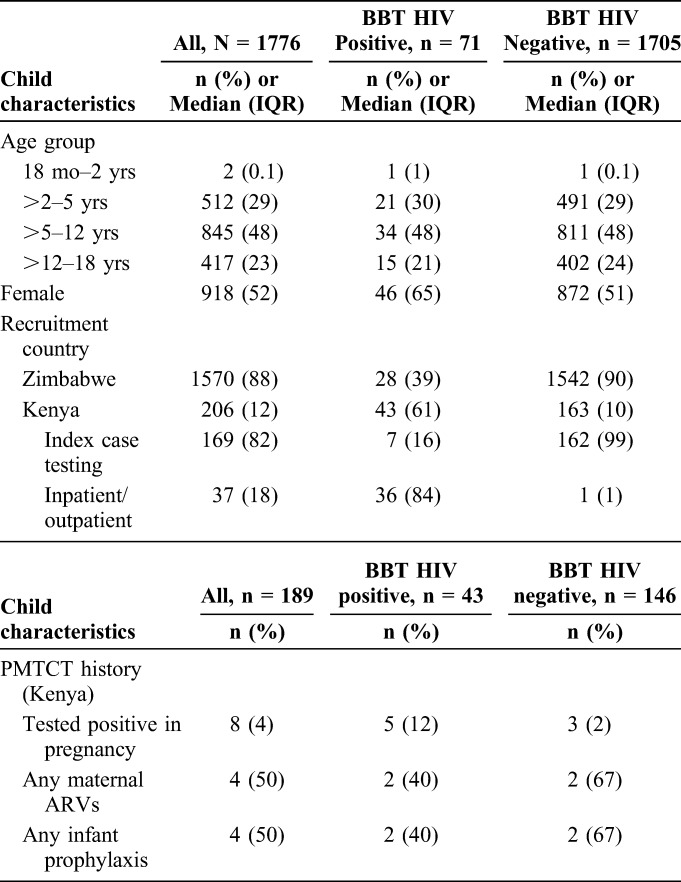
Sociodemographic Characteristics

## RESULTS

### Demographics

Overall, 1776 children were enrolled; 1570 (88%) from Zimbabwe and 206 (12%) from Kenya. The median age was 7.3 years (IQR: 4.7–11.6); 2 (0.1%) were 18 months −2 years; 512 (29%) were >2–5 years; 845 (48%) were >5–12 years; and 417 (23%) were >12–18 years. Overall, 918 (52%) were female. Among Kenyan children, 169 (82%) were identified via index case testing and 37 (18%) in outpatient clinics and inpatient wards (Table 1).

#### OMT Sensitivity and Specificity

Among 71 children positive by BBT, 71/71 (sensitivity: 100.0% [97.5% CI: 94.9% to 100.0%]) were positive by OMT. Among 1705 children negative by BBT, 1703/1705 (specificity: 99.9% [95% CI: 99.6% to 100.0%]) were negative by OMT. In the 1570 Zimbabwean participants, the PPV was 93.3% (95% CI: 77.9% to 99.2%) and the NPV was 100.0% (97.5% CI: 99.8% to 100.0%).

In Zimbabwe, 2 children who initially tested BBT-negative and OMT-positive were retested within 1 week to confirm HIV status because of suggestive clinical presentation and history; both were confirmed positive. A 9-year-old was confirmed positive by ELISA. A 2-year-old was confirmed positive by First Response and CHEMBIO. Excluding these 2 children, the sensitivity and specificity of OMT compared with those of BBT were each 100% (97.5% CI: 94.9% to 100% and 99.8% to 100%, respectively) (Table 2).

**TABLE 2.**
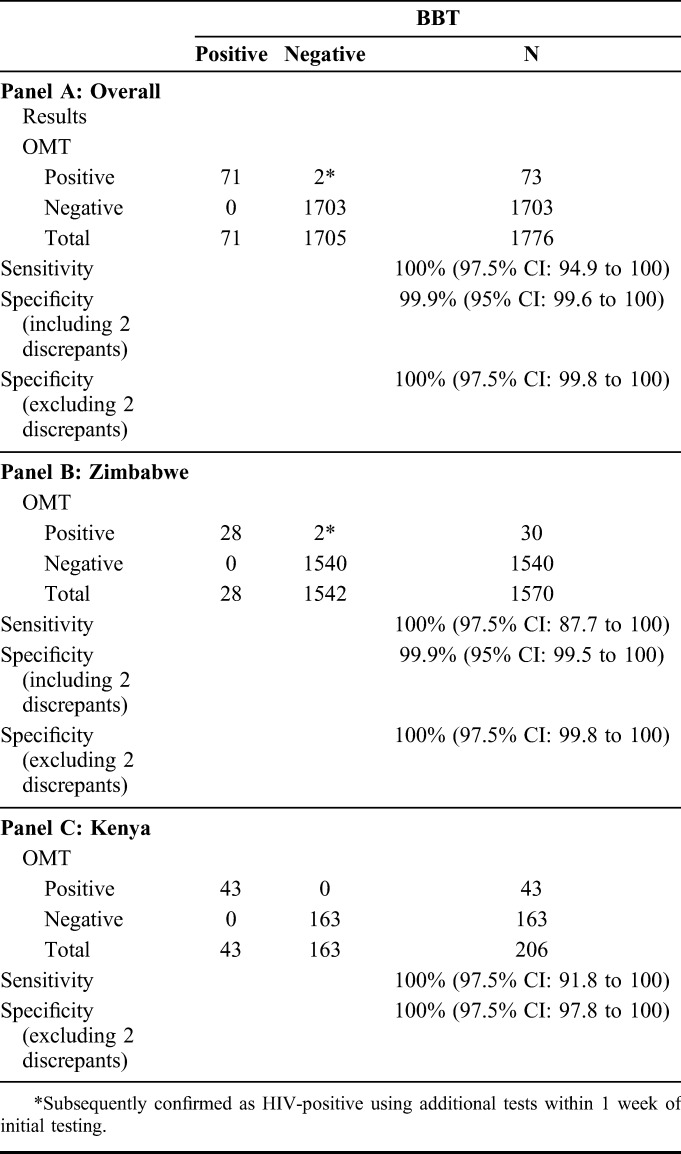
Performance of OMT vs BBT for HIV Diagnosis Overall and Stratified by Site

#### Stability of Visual Results (Kenya)

Among 43 children with positive OMT at 20 minutes, 43 (100%) had positive OMT at 40 minutes. Among the 163 children with negative OMT at 20 minutes, 163 (100%) had a negative OMT at 40 minutes. Using result interpretation pictures from the manufacturer, among 43 positive OMT results, 26 (60%) and 29 (67%) were strongly positive at 20 and 40 minutes, respectively. Three reads that were weakly positive at 20 minutes were strongly positive by 40 minutes; the remaining 14 (33%) were weakly positive at both 20 and 40 minutes.

## DISCUSSION

In this cross-sectional study of children aged 18 months to 18 years, we found that OMT had excellent sensitivity and specificity. When compared to the Kenyan and Zimbabwean national algorithms, OMT did not miss any positive children. These data suggest that OMT is valid for HIV diagnosis in this age range.

As with other antibody tests, OMT is inappropriate as a diagnostic test for children under 18 months due to the presence of maternal antibodies.^18^ In adults, antibody-based tests have limitations due to a long window period, which may lead to failure in detecting recent HIV infection.^19^ However, this is less of a concern among older children and younger adolescents who, if infected, are likely to have long-standing HIV, acquired perinatally.

Our results provide evidence for wider use of OMT for pediatric testing. Current testing approaches to identify children include index-linked testing, provider-initiated testing and counseling, targeted testing in health facilities, and community-based testing.^6,7,20–26^ Outpatient provider-initiated testing and counseling can identify children earlier in disease progression^27^; however, achieving high coverage is challenging^21^ because of high client volume and workload for limited numbers of HCWs.^28^ In resource-limited settings, scaling up testing will require simultaneously increasing coverage and minimizing costly components of testing, including HCW time.^29,30^ The ease and safety of OMT presents a potential opportunity for task-shifting from HCWs to lay providers, as was done in this study, or to caregivers to overcome human resource constraints. It is also important to note that the time to perform OMT is also similar to that required for BBT. Future research is needed to explore the acceptability and feasibility of OMT by caregivers and HCWs in facility and community settings.

A 2012 systematic review comparing OMT with whole blood specimens reported a pooled sensitivity of 98.0% and specificity of 99.7% for OMT.^10^ Despite this, the concentration of antibodies in oral fluid is lower than in blood and typically wanes during HIV treatment.^31,32^ Previous studies in Zimbabwe have confirmed that OMT has suboptimal sensitivity in ART-experienced children.^29,33^ WHO has issued warnings, advocating that rapid diagnostic tests not be used among ART-experienced adults; similar warnings seem warranted in children. Therefore, it is critical to avoid use of OMT by ART-experienced patients, either to confirm being “cured” of HIV or when reinitiating HIV care.^34^ Our study included an entirely ART-naive pediatric population and observed no false-negative results. In 2 cases, children were negative by BBT and positive by OMT and were confirmed HIV-positive upon repeat testing. This suggests slightly better detection by OMT than BBT in our study; it is unclear why we observed this counterintuitive finding.

Our study's strengths include a large sample of ART-naive, HIV-positive children to inform precise estimates of sensitivity. In addition, data from Kenya and Zimbabwe provided similar results. OMT results were compared with routine, field-based BBT according to national algorithms, which provides an apt comparison with standard-of-care tests and provides useful public health information. Limitations include that OMT result interpretation was not blinded in Kenya, which may have influenced result interpretation. National algorithms between the 2 countries differed slightly, so the “reference standard” was not the same in both countries. However, in both cases, the algorithms are those used for national guidelines. Consequently, our findings demonstrate the performance of OMT against the standard-of-care and are therefore generalizable in these settings. Although the BBT in this study was not ELISA or polymerase chain reaction (PCR) based, OMT has previously been compared with these more sensitive lab-based tests to inform Food and Drug Administration approval and WHO endorsement for use in adults.^11,31^ An additional limitation is that in our study we did not have any inconclusive test results. Procedures on how to report and manage inconclusive test results must be put in place.

## CONCLUSIONS

OMT is highly sensitive and specific in children and adolescents. This is consistent with findings from studies in adult populations. Policymakers and regulators should consider expanding the age at which OMT may be used to include children over 18 months.
